# Urban and transport planning, environmental exposures and health-new concepts, methods and tools to improve health in cities

**DOI:** 10.1186/s12940-016-0108-1

**Published:** 2016-03-08

**Authors:** Mark J. Nieuwenhuijsen

**Affiliations:** Center for Research in Environmental Epidemiology (CREAL), Barcelona, Spain; Universitat Pompeu Fabra (UPF), Barcelona, Spain; Centro de Investigación Biomédica en Red de Epidemiología y Salud Pública (CIBERESP), Madrid, Spain

## Abstract

**Background:**

The majority of people live in cities and urbanization is continuing worldwide. Cities have long been known to be society’s predominant engine of innovation and wealth creation, yet they are also a main source of pollution and disease.

**Methods:**

We conducted a review around the topic urban and transport planning, environmental exposures and health and describe the findings.

**Results:**

Within cities there is considerable variation in the levels of environmental exposures such as air pollution, noise, temperature and green space. Emerging evidence suggests that urban and transport planning indicators such as road network, distance to major roads, and traffic density, household density, industry and natural and green space explain a large proportion of the variability. Personal behavior including mobility adds further variability to personal exposures, determines variability in green space and UV exposure, and can provide increased levels of physical activity.

Air pollution, noise and temperature have been associated with adverse health effects including increased morbidity and premature mortality, UV and green space with both positive and negative health effects and physical activity with many health benefits. In many cities there is still scope for further improvement in environmental quality through targeted policies. Making cities ‘green and healthy’ goes far beyond simply reducing CO2 emissions. Environmental factors are highly modifiable, and environmental interventions at the community level, such as urban and transport planning, have been shown to be promising and more cost effective than interventions at the individual level. However, the urban environment is a complex interlinked system.

Decision-makers need not only better data on the complexity of factors in environmental and developmental processes affecting human health, but also enhanced understanding of the linkages to be able to know at which level to target their actions. New research tools, methods and paradigms such as geographical information systems, smartphones, and other GPS devices, small sensors to measure environmental exposures, remote sensing and the exposome paradigm together with citizens observatories and science and health impact assessment can now provide this information.

**Conclusion:**

While in cities there are often silos of urban planning, mobility and transport, parks and green space, environmental department, (public) health department that do not work together well enough, multi-sectorial approaches are needed to tackle the environmental problems. The city of the future needs to be a green city, a social city, an active city, a healthy city.

**Electronic supplementary material:**

The online version of this article (doi:10.1186/s12940-016-0108-1) contains supplementary material, which is available to authorized users.

## Background

Cities have long been known to be society’s predominant engine of innovation and wealth creation, yet they are also its main source of crime, pollution, and disease [1]. Bettencourt and colleagues [1] showed that processes relating urbanization to economic development and knowledge creation are very general, being shared by all cities belonging to the same urban system and sustained across different nations and times but that there are efficiencies of scale; quantities reflecting wealth creation and innovation have increasing returns, whereas those accounting for infrastructure show economies of scale. Recent estimates show that 60–80 % of final energy use globally is consumed by urban areas [2] and more than 70 % of global greenhouse gas emissions are produced within urban areas [3]. As a result, also environmental pollution increases with urbanization.

Lamsal and colleagues [4] found that urban NO_2_ pollution, like other urban properties, is a power law scaling function of the population size: NO_2_ concentration increases proportional to population raised to an exponent. The value of the exponent varies by region from 0.36 for India to 0.66 for China, reflecting regional differences in industrial development and per capita emissions. Fragkias and colleagues [5] found a near-linear relationship between population size and carbon emissions suggests that large urban areas in the U.S. are only slightly more emissions efficient than small ones. For each year in their sample, variation in population size across cities in the U.S. urban system explained approximately 70 % of the variation of CO2 emissions.

Already in 1973, Oke [6] described the relation between population and urban heat island effect. The high density of buildings and roads can cause so-called urban heat islands defined as built up areas that are hotter than nearby rural areas [7]. Fuller and colleagues [8] showed that in Europe green space coverage increases more rapidly than city area, but that a decline in green space availability per capita accelerates with increasing population density, suggesting that access to green space could decline rapidly as cities grow, increasing the geographical isolation of people from opportunities to experience nature.

In cities, environmental exposures such as air pollution [9, 13] temperature [14, 15] and noise [16] have been associated with adverse health effects, while ultraviolet radiation (UVR) [17] and green space [18, 20] have been associated with both positive and negative health effects, and are therefore important to measure and control.

Today, more than two thirds of the European population lives in urban areas and this share continues to grow. The development of our cities will determine the future economic, social and territorial development of the European Union [21]. Urban sprawl and the spread of low-density settlements is one of the main threats to sustainable territorial development; public services are more costly and difficult to provide, natural resources are overexploited, public transport networks are insufficient and car reliance and congestion in and around cities are heavy. Although air pollution decreased over the last decades in North American and European cities, more than 80 % of the population in the WHO European Region lives in areas with levels of ambient particulate matter (PM) exceeding WHO Air Quality Guidelines. The exposure to traffic noise is increasing as a result of continuing urbanization and rising traffic volumes, and around 20 % of the Europeans are regularly exposed to noise exceeding WHO guidelines [http://ec.europa.eu/environment/noise/health_effects.htm].

**Fig. 1:**
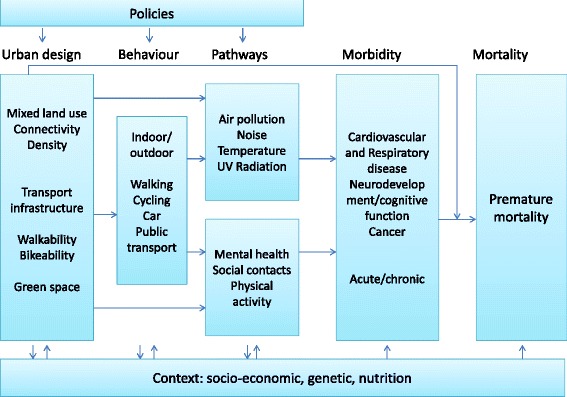
Conceptual framework for the relation between urban and transport planning, environmental exposures and health

In a seminal paper, Rydin and colleagues [22] provided an analysis of how health outcomes can be improved through modification of the physical fabric of towns and cities and discussed the role that urban planning can have in delivering health improvements. The work began from the premise that cities are complex systems, with urban health outcomes dependent on many interactions and feedback loops, so that prediction within the planning process is fraught with difficulties and unintended consequences are common. They provided, amongst others, separate examples on built environment and physical activity, green space and urban heat islands. Here we expand the work by Rydin and colleagues [22] on urban design, environmental exposures and health, evaluate the linkages and highlight the large exposure variation that exists within cities. The focus here is on cities in higher income countries, but applicable to those in low and middle income countries. The aim is to provide a narrative towards new insights and possible solutions for the current environmental and health challenges in cities, focusing on the links between built environment, environmental exposure and health and identifying new concepts, methods and tools to inform science and policies (Fig. 1).

## Methods

### Search strategy and selection criteria

We searched PubMed, Web of Science and Science Direct, and references from relevant articles in English language from Jan 1, 1980, to Oct 1, 2014, using the search terms: “city”, “urban” in combination with “air pollution”, “noise”, “temperature”, “UV”, “green space”, “heat island”, “carbon emissions”, “built environment”, “walkability”, and/or “mortality”, “respiratory disease”, “cardiovascular disease”, “hypertension”, “blood pressure”, “annoyance”, “cognitive function”, “reproductive outcomes” following an initial rapid review of the literature of the topic area and the author’s knowledge We focused on systematic reviews, meta-analyses and articles published in the past 5 years; however, we used older articles if they represent seminal research or are necessary to understand more recent findings.

## Results and discussion

### Linking urban planning indicators, environmental exposure and personal behaviour

Considerable variation exists in environmental and personal exposure to air pollution, noise, temperature, UV and green space within cities largely due to built environment and personal behaviour and an interaction between the two. Traffic indicators such as distance to major roads, surrounding road length, and traffic density, household density, industry and natural and green space explain a large proportion of the variability of air pollution within urban areas [23, 24]. For example, average concentrations of air pollutants are generally considerably higher at street locations compared to urban background with average ratios of 1.63 for NO_2_ and 1.93 for NOx [25] and 1.14, 1.38, 1.23 and 1.42 respectively for PM_2.5_, PM_2.5_ absorbance (soot), PM10 and PMcoarse [26] in Europe, resulting in considerable variation in air pollution levels within cities. Also the levels of ambient noise are associated with building density, road network, traffic flow, speed and load, junctions, acoustics and meteorological conditions in cities [27, 29] The L50 noise levels (total data) range from about 54 dBA (in acoustic shadows) in residential tertiary streets up to 74 dBA on the high traffic roads [28]. Generally there is low to moderate correlation between air pollution and noise. Foraster and colleagues [27] found a correlation of 0.62 between NO2 and noise (L24h). However, the correlation differed across the urban space, with lower correlations at sites with higher traffic density and in the modern downtown [27].

The urban heat island effect is often observed where open, wooded or green areas have been replaced by concrete and asphalt. It depends e.g. on population density, vegetation, urban design and albedo effects and can result in temperature differences between urban and adjacent rural areas of up to 3–5 °C [30–32]. Petralli and colleagues [32] found that intra-urban variability of summer values was almost 3 °C both in minimum and maximum air temperature. The amount of green space varies considerably between and within cities with green space coverage, averaging 18.6 % in European cities and ranging from 1.9 to 46 % [8]. A reduction of personal exposure to air pollution has been observed in areas with more green space [33], while vegetation has been suggested to reduce air pollution levels, and temperature [34, 35, 36], and vegetation (trees, plants) and soil may have an impact on the sound level [37–41]. Frank and colleagues [42] evaluated the association between a single index of walk ability that incorporated land use mix, street connectivity, net residential density, and retail floor area ratios, with health-related outcomes in King County, Washington. They found a 5 % increase in walkability to be associated with a per capita 32.1 % increase in time spent in physically active travel, a 0.23-point reduction in body mass index, 6.5 % fewer vehicle miles traveled, 5.6 % reduction in oxides of nitrogen (NOx) emitted, and 5.5 % reduction in volatile organic compounds (VOC) emitted. In general, land use measures such as density, connectivity and land use mix, and travel policies and interventions to increase walking and cycling are consistently associated with higher public transport use, more walking, and less driving, but there are few studies on the relation with environmental exposures [43]. Furthermore, many built environment attributes are strongly associated with higher densities making it difficult to isolate their effects. Finally people spent a large proportion of their time indoors (80–90 %), which affects the levels and frequency of their exposure to environmental factors [44, 45]. For example, de Nazelle and colleagues [45] found that people in Barcelona spent 51 % at home, 33 % at work 6 % of their time in transit. Dadvand and colleagues [44] found large variability in personal UV exposure in cities, even though ambient levels show little variability, because of the variability in duration people spent outdoors.

### Health effects of environmental exposures

#### Single exposures

Recent studies have shown effects of long-term within city exposure to air pollution on mortality [10, 11], lung cancer [12], cardiovascular disease incidence [13], decreased lung function in children [46, 47], respiratory infections during early childhood [48] and low birth weight [49] confirming previous studies based on both within and between city exposure to air pollution [9, 50]. Furthermore, evidence is emerging for a role of air pollution in other diseases such as diabetes [51, 52]. Ambient particulate air pollution was ninth in the ranking of the Global Burden of Disease estimates in 2010 [53] contributing to an estimated 3–4 million premature deaths and is estimated to reduce life expectancy by almost 9 months on average in Europe [54].

Ambient noise has been associated with a range of different health outcomes including cardio-vascular mortality and morbidity [16, 55–57], annoyance and sleep disturbances [16, 58, 59] high blood pressure in children [60], cognitive effects in children [16, 61, 62] and reproductive outcome [63]. Cardiovascular effects by ambient noise have been shown to be independent of air pollution exposures [62, 64–66].

High and low ambient temperatures have been associated with mortality [15, 67], cardio respiratory morbidity [14, 68, 69], hospital admissions [70] and children’s health [71]. Specifically, the urban heat island effect contributed significantly to health impacts of the 2003 heat wave in Paris [72]. The temperature-morbidity relationship however may be somewhat confounded or modified by sociodemographic factors and air pollution [68].

Exposure UV radiation (UVR) is associated with both beneficial effects such as Vitamin D increase [73, 74] and negative effects such as DNA damage [74]. Lucas and colleagues [17] suggested there is a global disease burden attributable to exposure to UVR of around 50,000 deaths and 1.6 million DALYs specifically for cutaneous malignant melanoma, cortical cataracts of the eye, non-melanoma skin cancers, solar keratoses and pterygium. Furthermore, more recent work suggests that chronic (not intermittent) sun exposure is associated with a reduced risk of colorectal-, breast-, prostate cancer and Non Hodgkin’s Lymphoma [75], and auto immune diseases [76].

Green space has been associated with a number of beneficial health effects [19, 20] including on reduced mortality and increased longevity [18, 77, 78], cardiovascular disease [79, 80], people’s self-reported general health [81–83], mental health [84], children’s behavioral problems [85, 86], sleep patterns [87], recovery from illness [88], social contacts [82, 89], the microbiome [90] and birth outcomes [91]. Increased physical activity and social contacts, psychological restoration/stress reduction, and a reduction in pollutants such as noise and air pollution, and heat have been proposed as possible mechanisms for the health benefits of green space [19, 20, 82]. However it has also been associated with some negative effects such as increased risk for Lyme disease and skin cancer [19, 20].

Finally it is important to note that exposure levels and exposure response relationships may differ by gender, social economic and ethnic groups for the exposures above, which should be considered when evaluating the health impacts.

#### Multiple exposures

Generally the effects described above have been obtained through epidemiological studies which focused on a specific environmental exposure and a health outcome, adjusted for important confounders, and occasionally adjusted for environmental co-variates to assess if the effects were independent of each other or whether there was some modifying or mediation effect, for example in terms of air pollution and noise [62, 64–66], temperature and air pollution [92–94] and green space, noise and air pollution [95, 96]. In a novel approach, Dadvand and colleagues [97] extended previous analyses, suggesting that proximity to major roads is a risk for term low birth weight. They considered the mediating roles of air pollution, noise, heat, and road-adjacent trees in a cohort of births in Barcelona. Their analysis suggested that air pollution and heat jointly account for one-third of the measured association between road proximity and low birth weight. More than in prior analyses, they considered multiple potential exposures related to urban form [98]. The work provides more information on the potential pathways. It matters what specific pathways link urban design to health, as these pathways can inform the most effective interventions, allowing us to design and retrofit cities for health [98]. For example, mixed land use is thought to make cities more livable—decreasing the distance between home, work, and amenities. However, it is not known how much of the associated health benefit might be due to housing quality, access to healthy or unhealthy amenities, environmental exposures, or the modification of individual risk behaviors. An important question is whether we can continue to address each of these factors in isolation [98].

### The application of new concepts, methods and tools to provide new insights

More recently to get away from studying the “one exposure, one health outcome” associations, a new paradigm has been developed, the exposome. The paradigm envisages complex multi-level pathways and interactions with other environmental, socioeconomic, social, behavioral and life-style factors, and genetics. The exposome encompasses the totality of human environmental (i.e., non-genetic) exposures from conception onwards, complementing the genome [99, 100]. Therefore, it requires consideration of both the nature of those exposures and their changes over time [100]. The exposome comprises processes internal to the body such as metabolism, endogenous circulating hormones, body morphology, physical activity, gut microflora, inflammation, lipid peroxidation, oxidative stress and ageing. Secondly, there is the extensive range of specific external exposures which include air pollution, infectious agents, chemical contaminants and environmental pollutants, diet, lifestyle factors (e.g. tobacco, alcohol), occupation and medical interventions. Thirdly, the exposome includes the wider social, economic and psychological influences on the individual, for example: social capital, education, financial status, psychological and mental stress, urban–rural environment and climate [100]. The dynamic nature of the exposome presents one of the most challenging features of its characterization. Only because of the increased use of new technologies including geographical information systems (GIS), sensors, remote sensing, OMICS technologies (e.g. transcriptomics, proteomics, metabolomics), combined with more traditional approaches has it become possible to start assessing the exposome and first attempts are being made in large European projects such as HELIX (http://www.projecthelix.eu/) [101], EXPOsOMICs (http://www.exposomicsproject.eu/) and HEALS (http://www.heals-eu.eu/).

The assessment of external environmental exposures in cities has often been a limiting factor in this type of research, but novel technologies may bring great advancements. Relatively cheap sensors are becoming available nowadays to measure environmental exposures such as air pollution [102], noise [28] and temperature [32] and can be placed in various locations in a city to capture the within city variation in exposure. Furthermore also satellite data can now be used to capture within city variation in air pollution [103], temperature [72, 97, 104, 105], and green space [85, 95]. Also the use of new technologies including smartphones, other GPS devices and small sensors can improve personal assessment of exposure by obtaining information on the location and mobility of a person, environmental exposure level information and physical activity levels [102, 106–113]. Many people in high income countries nowadays have smartphones which with the use of Apps can provide information to characterize exposure [45, 112, 114, 115]. The smartphone data can be used to show objectively where people spend their time, and therefore which level of exposure they may experience, when overlaid with exposure maps [45] or connected to pollution sensors [115]. Furthermore, the combination of assessment of personal air pollution concentrations and physical activity provides the opportunity to estimate the inhaled dose, which may be a better measure than exposure [45, 116, 117]. For example, in Barcelona de Nazelle and colleagues [45] found using modeled NO_2_ data that, on average, time at home, which represented 51 % of people’s time in a day, and similarly 54 % of daily time weighted exposures, accounted for 40 % of individuals’ total inhaled dose. Time at work, 33 % of people’s daily activity, led to 29 % daily time weighted exposures and 28 % of daily inhaled NO_2_. In reverse, volunteers only spent 6 % of their time in transit, yet this microenvironment contributed to 11 % of time weighted exposures in a day, and 24 % of daily inhaled NO_2_. Also in Barcelona using a Smartphone and a personal sensor measuring black carbon, Nieuwenhuijsen and colleagues [115] showed travelling routes and varying black carbon levels along the route, with the highest levels of black carbon during commuting, lower levels at school and the lowest level at home. Besides measuring exposures, other sensors worn personally can obtain information on health and physiological parameters and thereby obtain continuously and simultaneously information on environmental exposures and health [29, 112, 118, 119, 120]. This type of work also contributes to “smart cities” which are cities that use digital technologies to enhance performance and wellbeing, to reduce costs and resource consumption, and also to engage more effectively and actively with its citizens.

The involvement of a larger proportion of the population in cities through citizens science or the new citizens observatories that are being established to obtain more information on our environment, may offer greater opportunities for data collection [121–123]. Citizen science refers to the engagement of the general public in scientific research activities in which citizens actively contribute to science, be it through their intellectual inputs, knowledge or tools and resources. Citizens observatories can be defined as communities where citizens observe and try to understand environment-related problems, and more particularly assess, report and comment on them. Involving citizens on-site at a local level by developing knowledge pools, and obtaining and using their knowledge, will help to create an atmosphere of active participation and generate a sustainable movement that can build over time and lead to empowerment in environmental governance [124–126]. Citizens can use the information to make changes themselves or take it to policy makers to have them make the changes.

### From insights to actions to impacts

Premature mortality and unhealthy life years due to the environment is largely preventable. High blood pressure, obesity and physical inactivity are among the leading risk factors of non-communicable diseases (NCDs) such as cardiovascular disease, type 2 diabetes, and chronic lung diseases, which are major causes of death in European countries [127]). Non-communicable diseases (cardiovascular and respiratory diseases, cancer and diabetes) account for some 86 % of disability adjusted life years (DALYs) in Europe [128], and an estimated impact of up to 7 % on a country’s GDP [129]. One in every six children has a neurodevelopmental disability [130]; childhood obesity is one of the most serious public health challenges of the 21st century with dramatic rises in Europe in recent decades [131]; the prevalence of immune system-mediated outcomes - such as asthma and respiratory infections - in children is more than 20 % in some countries [132]. To what extent morbidity and premature mortality could be attributed to the built environment and related environmental exposures is still to a large extent unclear, but the numbers above are sufficiently large to warrant further action, even if the contribution is only small.

Traditionally, successful prevention efforts are mainly focused on adult life style related factors. However, an accumulating body of evidence suggests that the prevention of NCDs should already start in the earliest phase of life [133–135]. The pathways underlying the observed associations may include developmental adaptations of cardiovascular, metabolic, respiratory and cerebral systems, in response to adverse exposures during critical fetal and childhood periods. These adaptations may shift developmental trajectories and lead to a higher susceptibility of development of NCDs in later life and to earlier ageing [101, 134, 135].

Environmental factors are highly modifiable, but evidence is needed to decide where and when to intervene. Particularly, environmental interventions at the community level, such as urban and transport planning [136–139], have been shown to be promising and more cost effective than interventions at the individual level [140]. For example, the ban in coal burning in Dublin reduced the air pollution levels and related respiratory and cardiovascular mortality by 10 to 15 % [141] and stronger legislation and improved technologies have led to decreased air pollution levels and improved life expectancy in the US [142].

**Fig. 2:**
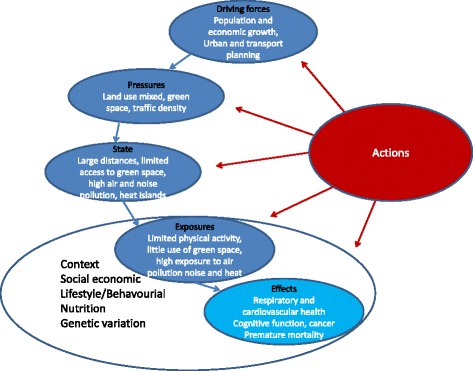
The modified DPSEA frame work for urban and transport planning, environmental exposures and health

However the urban environment is a complex interlinked system. Decision-makers need not only better data on the complexity of factors in environmental, personal behavioural and developmental processes affecting human health, but also enhanced understanding of the linkages to be able to know at which level to target their actions. The new concepts, method and tools described above could provide better insights. The modified D-P-S-E-E-A framework (driving forces, pressures, state, exposures, health effects and actions) may be helpful for policy and actions as it provides a logical chain of driving forces, pressures, exposures and their specific determinants and effects and also identifies specific areas that can be targeted for actions [143–145]. However, it may be limited because it may not include all the complexities and further work is needed on this. In cities, driving forces such as increased urban and population growth, the economic climate and cultural preferences have a profound effect on urban and transport planning and may result in pressures such as high car traffic density, limited green and public space areas and mixed land zones, loss of social capital and increases in (fast) food restaurants. This may result in a state e.g. high air pollution levels, reduced access to green space, larger distances to travel and poor food environment, with as consequence e.g. high exposures to air and noise pollution and heat, limited physical activity, limited social contacts/cohesion, a reduction of opportunities for active transportation, greater opportunities for fast food consumption leading to possible effects on respiratory and cardiovascular health, growth/obesity, and behavioral disorders/cognitive function. Finally, it is important to consider the context including socioeconomic position, social environment, life style/behavior, nutritional status and genetic may play a large role and modify the relationships (Fig. 2).

To inform any proposed policies and actions, health impact assessments have recently been conducted to take into account and quantify any potential health risks and benefits for different scenarios to evaluate the overall potential impacts of a proposed action, especially for actions that may involve multiple exposures and health effects [146, 147]. Insightful assessments have been conducted for cities in relation to transport policies quantifying both potential health risks and benefits in terms of e.g. physical activity, air pollution and accidents [148–152].

Making cities ‘green and healthy’ goes far beyond simply reducing CO_2_ emissions. A systemic approach to urban and transport planning, environmental and energy issues has to be adopted, as the many components of the natural ecosystem are interwoven with those of the social, economic, cultural and political urban system in a unique manner. A sustainable city must have attractive open public spaces and promote sustainable, inclusive and healthy mobility. Non-car mobility has to become more attractive and multimodal public transport systems favoured [21]. Initiatives like a car free Hamburg by 2034 should be encouraged and replicated [153].

Urban and transport planning therefore also plays a key role. While in cities there are generally silos of urban planners, mobility and transport, parks and green space, environmental department, (public) health department that do not work together well enough, multi-sectorial approaches are needed to tackle the problems. Furthermore work is needed to bring the various sectors together and to show that systemic approaches involving multiple sectors may have benefits for all, through direct and co-benefits of specific policies.

For example, some potential policies such as a reduction of car use by increasing public and active transportation [43, 149–152] and increasing green space areas [154, 155] have joint benefits in that they may not only reduce carbon emissions and environmental exposures such as air pollution, noise, and temperature (i.e. heat islands), but also increase physical activity, UV exposure, and social contacts and reduce stress, and thereby reduce morbidity and premature mortality [43]. Furthermore, physical activity in green spaces appears to have added benefits [156] and cyclists prefer to cycle through greener areas [157]. Furthermore they create co-benefits such as reduction in congestion.

## Conclusions

In conclusion, in this paper we have put cities in a wider context and made links between urban and transport planning, environment and health. We considered multiple environmental exposures identifying common determinants and linking the built environment, environmental concentrations, personal behavior and exposures and health. We provided a state of the art on the health effects of important environmental exposures in cities and provided a framework to link science and policies. Finally we proposed a range of new concepts, methods and tools such as the exposome, citizens science and citizens observatories, environmental, personal and remote sensing, and health impact assessment that can be employed to improve understanding and inform policies and actions. Further work is urgently needed to reduce the burden of disease related to the built environment and environmental exposures in cities and make cities a health promoting place. For this to happen we need collaboration between e.g. researchers and practioners in urban planning, mobility and transport engineering, architecture and landscape architecture, environmental science, behaviour, and public health. The city of the future needs to be a green city, a social city, an active city, a healthy city.

## Additional file

Additional file 1:
**Peer review reports.** (PDF 54 kb)
