# Supplementary material for: Urban and transport planning, environmental exposures and health-new concepts, methods and tools to improve health in cities
Source: Environ Health. 2016 Mar 8;15(Suppl 1):38. doi: 10.1186/s12940-016-0108-1 (PMC4895603; doi:10.1186/s12940-016-0108-1)
Supplement: Additional file 1: — Peer review reports. (PDF 54 kb) [file 12940_2016_108_MOESM1_ESM.pdf]

Reviewer 1

Dear Sotiris,

Please find below my review of the manuscript 1476-069X-14-S1-S14.

Review 1476-069X-14-S1-S14

The submitted manuscript "Urban planning, environmental exposures and health – new concepts, methods and tools to improve health in cites" summarizes/presents recent research, tools and methods which help clarify the complex linkages between built environment, environmental exposure and health. The manuscript is a comprehensive review about this topic and clearly sets out the issues involved illustrated by many recent and relevant examples from the literature. The author has managed to write a very useful overview paper for both the science community and policy makers to inform them about the most up-to-date developments in this rapidly changing field.

I have only a few minor comments to this well written paper and therefore have no hesitation to recommend for this manuscript to be accepted for publication.

***Minor comments*:**

Page 6, 1st paragraph: What is meant by "mix" in "connectivity and mix"?

Page 12, 1st paragraph: where does the "(see above)" refers to? Either explain or remove.

Separate from the review I have also found some minor grammatical errors, which I suspect your editor will pick out as well. For your information, I add them at the end of this email. I don't expect you to add those to the published review.

Minor edits (for editor only):

Page 3, 1st paragraph: In ".., whereas those accounting for infrastructure **display show** economies of scale" please remove either "display" or "show"

Page 4, 2nd paragraph: Change "amongst" to "amongst others" in "They provided, amongst, separate examples on built…."

Page 5, 2nd paragraph: Add ")" after "shadows" in "(in acoustic shadows"

Page 6, 2nd paragraph: Add a comma after "Furthermore" and change "disease" into "diseases"

Page 7, 3rd paragraph: Add "(UVR)" after "UV radiation"

Page 7, 3rd paragraph: Add a comma after "Furthermore"

Page 7, 4th paragraph: Add "with" in "Green space has been associated **with** a number of beneficial health effects"

Page 8, 1st paragraph: Remove "an" in "adjusted for **an** environmental co-variates", change "eachother" to "each other" and add "whether" to "…or **whether** there was some modifying.."

Page 11, 2nd paragraph: Remove "Furthermore" from start of first sentence

Page 13, 2nd paragraph: change "cyclist" to "cyclists"

Kees de Hoogh PhD

Environmental Exposure and Health Unit
Department of Epidemiology and Public Health
Swiss Tropical and Public Health Institute

Reviewer 2

The paper “Urban planning, environmental exposures and health-new concepts, methods and tools to improve health in cities” submitted for publication in the special issue on “Challenges and Opportunities for Urban Environmental Health and Sustainability” in the Environmental Health journal constitutes a very interesting reflection on the complex dynamics and relationships among human activities, exposures and effects in urban areas.
The author discusses the main health-relevant factors through a thorough and comprehensive review of the scientific literature. In this analysis the main environmental factors and health endpoints are identified as well as the methodologies to relate changes in environmental burden with health effects.

The main aim of the contribution is to present new insights and concepts that may help to review urban and transport planning in order to improve the living conditions and health of citizens in urban areas. This new insights and concepts are related to improved knowledge (mainly regarding the need to address the health response under an integrated multi-factor, dynamic approach) and the availability of new technologies really useful to gain a better understanding on people behaviour and mobility as well as intensive information on environmental levels (pollution, temperature, etc).

The work is certainly relevant and includes interesting ideas. The author claims that this contribution provides a framework to link science and policies. However, this is not clear in my opinion and I see a clear potential for improvement at this particular point. I think that the robustness of the study and the relevance of the recommendations and relevance of potential courses of action derived from the study may be improved by further elaboration and integration of the concepts being discussed. Despite minor errors and typos, my main suggestions and concerns are as follows:

Introduction
The introductory section may be shortened since is somehow redundant with the “Linking urban planning indicators, environmental exposure and personal behaviour” and the “Health effects of environmental exposure”. I understand the author is trying to provide a very general view of urban environmental issues and needs to rely on aggregated, simple indexes and concepts, but some of them may be too ambiguous and/or misleading. For instance, he suggests that NO_2 pollution (he doesn’t specify whether he refers to emissions or concentration, and in that case what kind of temporal or spatial aggregation level) can be explained by population size. Similarly he states that CO_2 emissions are proportional to population although he also highlights the influence of other factors such as public transport systems or urban sprawl which is to some point, contradictory. I think that this section may definitively improve by trying to integrate a bit more the information to extract more compact and general trends/conclusions and avoid very specific and arguable indexes that may be totally dependent on the specific features of the urban areas they were elaborated from.
From the last paragraph in page 3 it can be inferred that green spaces may have negative effects. The author may be referring to the influence of vegetation on pollutant dispersion under very specific conditions, but this idea is neither further elaborated nor used in the following.

Search strategy and selection criteria
Although the review process made is impressive, the scientific literature regarding the terms listed is really wide. Some more information about the rationale and some details

regarding the methodology for selection of research articles may help to understand the work done.

Health effects of environmental exposure

The idea of providing potential pathways to link urban design to health is particularly interesting. Learning from successful measures or specific urban layouts may be an effective way to improve public health. However, how planning and urban features may help to retrofit cities for health remains unclear. I think that this section would benefit from a deeper reflection on how these principles may be applied to cities that were planned and build hundreds of years ago and also considering that any measure intended to change mobility patterns or urban infrastructure usually entails significant investments and/or socio-political costs.

From the discussion in this section the potential contribution of citizens observatories and citizen science to alleviate urban pollution / health impacts in urban areas not obvious. It would be interesting if the author could further elaborate on this.

From insights to actions to impacts

Although the contribution focuses on providing a review of new insights and methods and not that much on proposing particular actions to achieve healthier cities, I miss a stronger connexion with the previous discussion. The author states that switching from coal to cleaner fuels in the residential sector or increase public transport and green areas are effective options for a healthier environment. I don’t think anyone may disagree but to what extent this kind of recommendation relies on the insights and concepts introduced before is rather unclear. I would strongly encourage the author to try to expand the discussion regarding the DPSEEA (in the text, DPSEA in Figure 2 caption) framework for a more coherent integration of the different sections of the manuscript and the final remarks and suggestion what is too weak in its previous state.

Reviewer 3

This is an interesting and provocative paper that integrates the knowledge we have on various environmental risk factors at urban level and proposes new approaches for research and intervention. The paper is well written and covers a large part of the issues. It merits publication and it will enrich the quality of the volume.

The only concern I have is related to the link between environmental factors at urban level and socioeconomic status/ poverty issues. This is a topic that it is controversial in the scientific literature as positive and negative associations between air pollution, noise (etc) exposures and SES have been found. In addition low SES seems to be an effect modifier. In addition, the emerging problem of large migration into the cities of different ethnic groups should be considered. All these aspects are not mentioned in the paper and it is unclear how the new vision of urban planning could be able to address social inequality and racial issues. The paper needs to be expanded on that to some extent.
